# Striatal Astrocytes Act as a Reservoir for L-DOPA

**DOI:** 10.1371/journal.pone.0106362

**Published:** 2014-09-04

**Authors:** Masato Asanuma, Ikuko Miyazaki, Shinki Murakami, Francisco J. Diaz-Corrales, Norio Ogawa

**Affiliations:** Department of Brain Science, Okayama University Graduate School of Medicine, Dentistry and Pharmaceutical Sciences, Okayama, Japan; Prince Henry’s Institute, Australia

## Abstract

L-DOPA is therapeutically efficacious in patients with Parkinson’s disease (PD), although dopamine (DA) neurons are severely degenerated. Since cortical astrocytes express neutral amino acid transporter (LAT) and DA transporter (DAT), the uptake and metabolism of L-DOPA and DA in striatal astrocytes may influence their availability in the dopaminergic system of PD. To assess possible L-DOPA- and DA-uptake and metabolic properties of striatal astrocytes, we examined the expression of L-DOPA, DA and DAT in striatal astrocytes of hemi-parkinsonian model rats after repeated L-DOPA administration, and measured the contents of L-DOPA, DA and their metabolite in primary cultured striatal astrocytes after L-DOPA/DA treatment. Repeated injections of L-DOPA induced apparent L-DOPA- and DA-immunoreactivities and marked expression of DAT in reactive astrocytes on the lesioned side of the striatum in hemi-parkinsonian rats. Exposure to DA for 4****h significantly increased the levels of DA and its metabolite DOPAC in cultured striatal astrocytes. L-DOPA was also markedly increased in cultured striatal astrocytes after 4-h L-DOPA exposure, but DA was not detected 4 or 8****h after L-DOPA treatment, despite the expression of aromatic amino acid decarboxylase in astrocytes. Furthermore, the intracellular level of L-DOPA in cultured striatal astrocytes decreased rapidly after removal of extracellular L-DOPA. The results suggest that DA uptaken into striatal astrocytes is rapidly metabolized and that striatal astrocytes act as a reservoir of L-DOPA that govern the uptake or release of L-DOPA depending on extracellular L-DOPA concentration, but are less capable of converting L-DOPA to DA.

## Introduction

L-DOPA therapy is used in Parkinson’s disease (PD) to replenish with dopamine (DA) in damaged dopaminergic neural system. Although DA neurons are severely degenerated, L-DOPA is therapeutically efficacious in patients with PD and animal models of this disease. Where and how is DA synthesized in patients treated with L-DOPA? It is thought that L-DOPA is uptaken by serotonergic neurons or surviving sprouted dopaminergic neurons [1], [2], [3], [4] to synthesize DA, because serotonin neurons possess a common monoamine-synthesizing enzyme; aromatic amino acid decarboxylase (AADC). Previous reports demonstrated that repeated administrations of L-DOPA markedly increased DA turnover on the lesioned side of the striatum in hemi-parkinsonian rats [5], [6].

On the other hand, the expression of membrane receptors, channels and transporters in astrocytes is thought to be involved in neuron-astrocyte communication [7], [8], [9], [10]. Especially, neutral amino acid transporter (LAT) and DA transporter (DAT) are expressed in cortical and hippocampal astrocytes [11], [12], [13], [14], [15]. Based on the above background, we postulated that the uptake and metabolism of L-DOPA and DA in striatal astrocytes influences their availability in the dopaminergic system of PD patients. To assess L-DOPA- or DA-uptake property of striatal astrocytes in damaged dopaminergic neuronal system, we examined the expression of L-DOPA, DA and DAT in striatal astrocytes in a hemi-parkinsonian animal model injected repeatedly with L-DOPA and in cultured striatal astrocytes. To clarify the uptake, metabolism and release of L-DOPA and DA in striatal astrocytes, the contents of L-DOPA, DA and their metabolite in cultured astrocytes and in culture media were measured after L-DOPA/DA treatment.

## Materials and Methods

### Animals

All animal procedures described in our experiments were in strict accordance with the Guideline for Animal Experiments of Okayama University Advanced Science Research Center, and were approved by the Animal Care Use Committee of Okayama University Advanced Science Research Center. Special care was taken to minimize the number of animals used in this research. Male Sprague-Dawley rats and timed pregnant Sprague-Dawley rats were purchased from Charles River Japan, Inc. (Yokohama, Japan).

### L-DOPA treatment of hemi-parkinsonian rats

Unilateral nigrostriatal dopaminergic lesions were generated using 6-hydroxydopamine (6-OHDA) and the method described previously with some modification [6]. Briefly, 6-OHDA hydrobromide (Sigma-Aldrich Corp., St. Louis, MO; 4 µg in 2 µl saline containing 0.1% ascorbate) was injected at two sites (4 µg/site) in the right medial forebrain bundle of 9-week-old male Sprague-Dawley rats under pentobarbital anesthesia (50 mg/kg, i.p.) at the following coordinates: A −1.0 mm, L +1.8 mm, V +8.5 mm; A −1.4 mm, L +1.5 mm, V +8.0 mm from the bregma, according to the atlas of the rat brain [16] (upper incisor bar set 5.0 mm above the interaural line). Sham-operated control rats were injected with the same volume of saline containing 0.1% ascorbate. Apomorphine-induced rotation test was performed 2 weeks after 6-OHDA injection to confirm induction of the lesion by 6-OHDA. Approximately 60% of 6-OHDA-lesioned rats showed rotation behavior after apomorphine injection (0.1 mg/kg, s.c.). Rats that exhibited asymmetric rotation behavior towards the contralateral side of >50 turns/10 min after apomorphine injection were selected as hemi-parkinsonian models for the subsequent study.

Three weeks after 6-OHDA lesioning (at 1 week after apomorphine test), the hemi-parkinsonian rats were injected intraperitoneally with L-DOPA (50 mg/kg/day)/carbidopa (5 mg/kg/day) or the same volume of vehicle 0.25% methylcellulose once a day for 7 days. At 3 h after the final administration, the rats were deeply anesthetized and transcardially perfused with ice-cold saline followed by a fixative containing 4% paraformaldehyde (PFA), 0.35% glutaraldehyde in 0.1 M phosphate buffer (PB; pH 7.4). The brains were immediately removed from the skull and post-fixed for 24 h in a fixative containing 4% PFA in 0.1 M PB. After cryoprotection for 72 h in 15% sucrose in 0.1 M PB with 0.1% sodium azide, the brains were frozen and cut into 20-µm coronal sections on a cryostat. Finally, the brain sections containing the mid-striatum (+2.20 to +0.26 mm from the bregma) were stored in 10 mM phosphate-buffered saline (PBS) with 0.1% sodium azide at 4°C until staining.

### Fluorescence immunohistochemistry of rat brain sections

The rat brain sections were incubated in 1% normal goat serum for 30 min at room temperature (RT), and then reacted with mouse anti-glial fibrillary acidic protein (GFAP) monoclonal antibody (dilution; 1∶10,000; Millipore, Temecula, CA, #MAB360) and either rabbit anti-L-DOPA polyclonal antibody (dilution; 1∶250; Abnova, PAB0031), rabbit anti-DA polyclonal antibody (dilution; 1∶200; Millipore, #AB122S) or rabbit anti-DAT polyclonal antibody (dilution; 1∶1,000; Millipore, #AB1591P) for 18 h at 4°C. The antibodies were diluted in 10 mM PBS containing 0.2% Triton X-100 (0.2% PBST). After washing in 0.2% PBST (5×5 min), the sections were reacted for 2 h at RT with fluorescence-conjugated secondary antibodies. The secondary antibodies used were goat anti-mouse IgG Alexa Fluor 594- or anti-rabbit IgG Alexa Fluor 488-conjugated antibody (dilution; 1∶1000; Molecular Probes, Eugene, OR) for GFAP/L-DOPA staining and goat anti-mouse IgG fluorescent isothiocyanate (FITC)-conjugated antibody (dilution; 1∶500; Millipore) and goat anti-rabbit IgG rhodamine-conjugated antibody (dilution; 1∶250; Millipore) for GFAP/DA or DAT staining.

### Cell culture

Primary striatal or mesencephalic cell cultures were prepared as descried previously [17] with minor modifications [18]. Briefly, the striatum and mesencephalon were dissected out from Sprague-Dawley rat embryos at 15 days of gestation. After treatment with trypsin, cells from the striatum or mesencephalon were incubated with 0.004% deoxyribonuclease I and 0.03% trypsin inhibitor (Sigma-Aldrich) at 37°C for 7 min. After centrifugation (420 *g*×5 min), the cell pellet was gently resuspended in 4 ml of Dulbecco’s Modified Eagle Medium (DMEM; Gibco BRL, Rockville, MD) supplemented with 10% fetal bovine serum, 4 mM L-glutamine, and 60 µg/ml kanamycin sulfate. The dissociated cells were plated at a density of 2×10^5^ cells/cm^2^ onto six-well culture plates or four-chamber glass culture slides coated with poly-D-lysine (Becton Dickinson Labware, Bedford, MA). To obtain neuron-enriched cultures, the medium was replaced with fresh medium supplemented with 2 µM cytosine-ß-D-arabinofuranoside (Ara-C) to inhibit the replication of non-neuronal cells, at 24 h after initial plating of dissociated cells, and then incubated for 5 more days. In order to obtain glial-enriched cell cultures (astrocytes), dissociated cells were incubated in the same culture medium for 5–7 days, then subcultured and plated at a density 4.1×10^4^ cells/cm^2^, and finally incubated for another 7 days. Over 95% of these cultured glial-enriched cells showed GFAP immunoreactivity.

**Figure 1:**
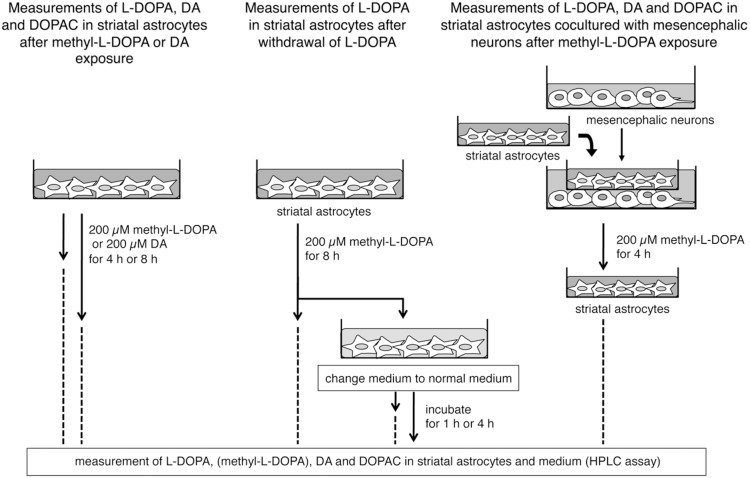
Schematic illustration of the protocols used for treatment of cultured striatal astrocytes for measurements of L-DOPA, DA and its metabolite after exposure to methyl-L-DOPA or DA (left, corresponding to Table 1), for measurements of L-DOPA after withdrawal of L-DOPA (middle, corresponding to Fig. 7), and for measurements of L-DOPA, DA and DOPAC in striatal astrocytes cocultured with mesencephalic neurons after methyl-L-DOPA exposure (right, corresponding to Table 2).

To measure monoamines and their metabolites in astrocytes, the striatal astrocytes were plated onto 6-well plates (Becton Dickinson) and grown in DMEM containing 10% FBS at a density of 6.4×10^4^ cells/cm^2^ for 7 days. Then, astrocytes were treated with 200 µM methyl-L-DOPA ester hydrochloride (methyl-L-DOPA; Sigma-Aldrich, D1507) or 200 µM DA and were incubated for 4 h or 8 h (Fig. 1, left). To examine the release of L-DOPA from astrocytes after withdrawal of L-DOPA from the culture media, the striatal astrocytes were treated with methyl-L-DOPA (200 µM) for 8 h and then were incubated in fresh culture medium for 1 h or 4 h after changing the medium (Fig. 1, middle).

For membrane-delimited coculture of striatal astrocytes with mesencephalic neurons, subcultivated striatal astrocytes were plated onto cell culture inserts for 6-well plates (0.4 µM membrane pore size; 4.2 cm^2^ of area; Becton Dickinson) at 1.2×10^5^ cells/cm^2^ on day 5, and were co-incubated with precultured mesencephalic neurons on poly-D-lysin-coated 6-well plates for 2 days, and then treated with methyl-L-DOPA (200 µM) for 4 h (Fig. 1, right).

The procedures used for each treatment are illustrated in Fig. 1. After the final treatment or incubation, astrocytes were thoroughly washed with PBS.

### Fluorescence microscopy

All slides prepared for immunofluorescence studies of rat brain sections and primary cultures were first observed under a fluorescence microscope (Olympus BX50-FLA, Tokyo, Japan) using a mercury lamp through a 470–490 nm or 530–550 nm band-pass filter to excite Alexa Fluor 488/FITC or Alexa Fluor 594/rhodamine, respectively. Light emitted from Alexa Fluor 488/FITC or Alexa Fluor 594/rhodamine was collected through 515–550 nm band-pass filter or 590 nm long-pass filter, respectively. Co-localization of GFAP-positive signals and L-DOPA-, DA- or DAT-positive signals on immunostained brain sections was confirmed by confocal laser-scanning microscopy (model LSM 510, Carl Zeiss, Jena, Germany). The 488 nm line of an argon-ion laser attenuated to 5% of the maximal intensity with a neutral density was used to excite Alexa Fluor 488/FITC. The 543 nm line of a helium-neon laser without attenuation was used to excite Alexa Fluor 594/rhodamine. Light emitted from Alexa Fluor 488/FITC or Alexa Fluor 594/rhodamine was collected through a 505–530 nm band-pass filter or 560 nm long-pass filter, respectively. Images were taken at ×400 magnification and recorded using the Windows-based Zeiss LSM software. Adobe Photoshop CS4 software (Adobe, Waltham, MA) was used for digital amplification of the images.

### RT-PCR

Neuron-enriched cells or glial-enriched cells (astrocytes) from the striatum or mesencephalon on the six-well culture plates were washed with 10 mM PBS, and the RNA was extracted by 800 µl TRIzol reagent (Invitrogen, Carlsbad, CA). All samples were treated with DNase I (Promega, Madison, MI). After completion of cDNA synthesis with total RNA (1 µg), Oligo(dT)_12-18_ primer (Gibco) and reverse transcriptase (Takara Bio Inc., Shiga, Japan), 10 µl of reverse transcriptase products were utilized for PCR in a total volume of 50 µl using 1.25 U AmpliTaq Gold (Perkin-Elmer, Branchburg, NJ) and specific primers for cDNA amplification of *rat DAT* (GenBank accession number #M80233: upper 5′-CTTCACCAGAGCCGTGGCATTGATGAC-3′ and lower 5′-TATTGTAACTGGAGAAGGCAATCAGC-3′) [19]. PCR was performed under the following conditions: denature at 95°C for 1 min, annealing at 58°C for 1 min, extension at 72°C for 2 min in a total of 40 cycles, and a final extension at 72°C for 7 min. In the next step, the PCR products (340 bp) were separated on a 2% agarose gel and visualized by ethidium bromide staining.

### Western blot analysis

Western blot analysis was performed as described previously [20], [21]. Briefly, cultured neuron-enriched cells or astrocytes from the striatum or mesencephalon on six-well culture plates were washed with 10 mM PBS, and then lysed in 150 µl of ice-cold RIPA buffer [1 mM PBS, pH 7.4, 1% Nonident P-40, 0.5% sodium deoxycholate, and 0.1% sodium dodecyl sulfate (SDS)] plus a protease inhibitor (0.1 mg/ml phenylmethylsulfonyl fluoride in isopropanol). After incubation on ice for 60 min, the homogenates were centrifuged (19,200×*g*, 20 min at 4°C), and then the supernatant of each sample was collected. The total protein concentration of the cell lysates was determined by the Lowry-based Bio-Rad DC protein assay kit (Bio-Rad, Richmond, CA) with bovine serum albumin as a standard. Samples were mixed with the sampling buffer (4% SDS, 0.02% bromophenol blue, 20% glycerol, and 10% 2-mercaptoethanol in 125 mM Tris-HCl, pH 6.8), and then boiled for 2 min. The proteins were separated through a 12.5% SDS-polyacrylamide gel (Bio-Rad Laboratories), and then electrophoretically transferred onto nitrocellulose membranes (Hybond-ECL; Amersham Biosciences, Buckinghamshire, UK).

The membranes were blocked in Tris-buffered saline with 0.1% Tween-20 (TBS-T) containing 5% non-fat milk powder at room temperature for 1 h. Then the blots were incubated with goat polyclonal anti-DAT antibody (1∶200, Santa Cruz Biotechnology, Santa Cruz, CA, #K-20), rabbit polyclonal anti-L-type amino acid transporter LAT1 antibody (dilution; 1∶100, Serotec, Oxford, UK, #AHP735), mouse monoclonal anti-4F2hc antibody (dilution; 1∶200, BD Transduction Laboratories, #611516) or rabbit polyclonal anti-aromatic L-amino acid decarboxylase (AADC) antibody (dilution; 1∶500, Protos Biotech Corporation, New York, NY, #CA201 bDCrab) at room temperature for 1 h. After washing with TBS-T (2×5 min), the blots were reacted with donkey anti-goat IgG (Millipore), donkey anti-rabbit IgG (Amersham Biosciences) or donkey anti-mouse IgG (Millipore) secondary antibody conjugated with horseradish peroxidase (dilution; 1∶2,000 or 5,000) at RT for 1 h. Specific signals of proteins were visualized by chemiluminescence using the ECL Western blotting detection system (Amersham Biosciences).

### Measurement of intracellular L-DOPA, DA and its metabolites

The striatal astrocytes treated with methyl-L-DOPA or DA were homogenized with 5 volumes of 200 mM ice-cold perchloric acid containing 10 mM EDTA. After centrifugation (11,750×*g*, 20 min at 4°C), the supernatant was filtered (0.45 µm) and then injected directly into a high-performance liquid chromatography with electrochemical detectors (HPLC-ECD, Tosoh Co., Tokyo) to measure the concentrations of L-DOPA, methyl-L-DOPA, DA and their metabolite DOPAC. The HPLC system consisted of a delivery pump (PX-8020, Tosoh Co.) and an analytical column (EICOMPAK SC-5ODS, 3.0 mm×150 mm, Eicom Co., Kyoto, Japan). An electrochemical detector (EC-8020, Tosoh Co.) with glassy carbon was used at a voltage setting of 700 mV and an Ag/AgCl reference electrode. A mobile phase containing 0.1 M citrate-sodium acetate buffer (pH 3.5), methanol (17% v/v), EDTA-2Na, and sodium 1-octanesulfonate was infused at a flow rate of 0.6 ml/min.

### Statistical analysis

Results are presented as mean ± SEM. Differences between groups were analyzed by one-way or two-way ANOVA followed by *post-hoc* Fisher’s PLSD test. A *P* value less than 0.05 denoted the presence of a statistically significant difference.

## Results

### Expression of L-DOPA in the striatal astrocytes in hemi-parkinsonian model rats

**Figure 2:**
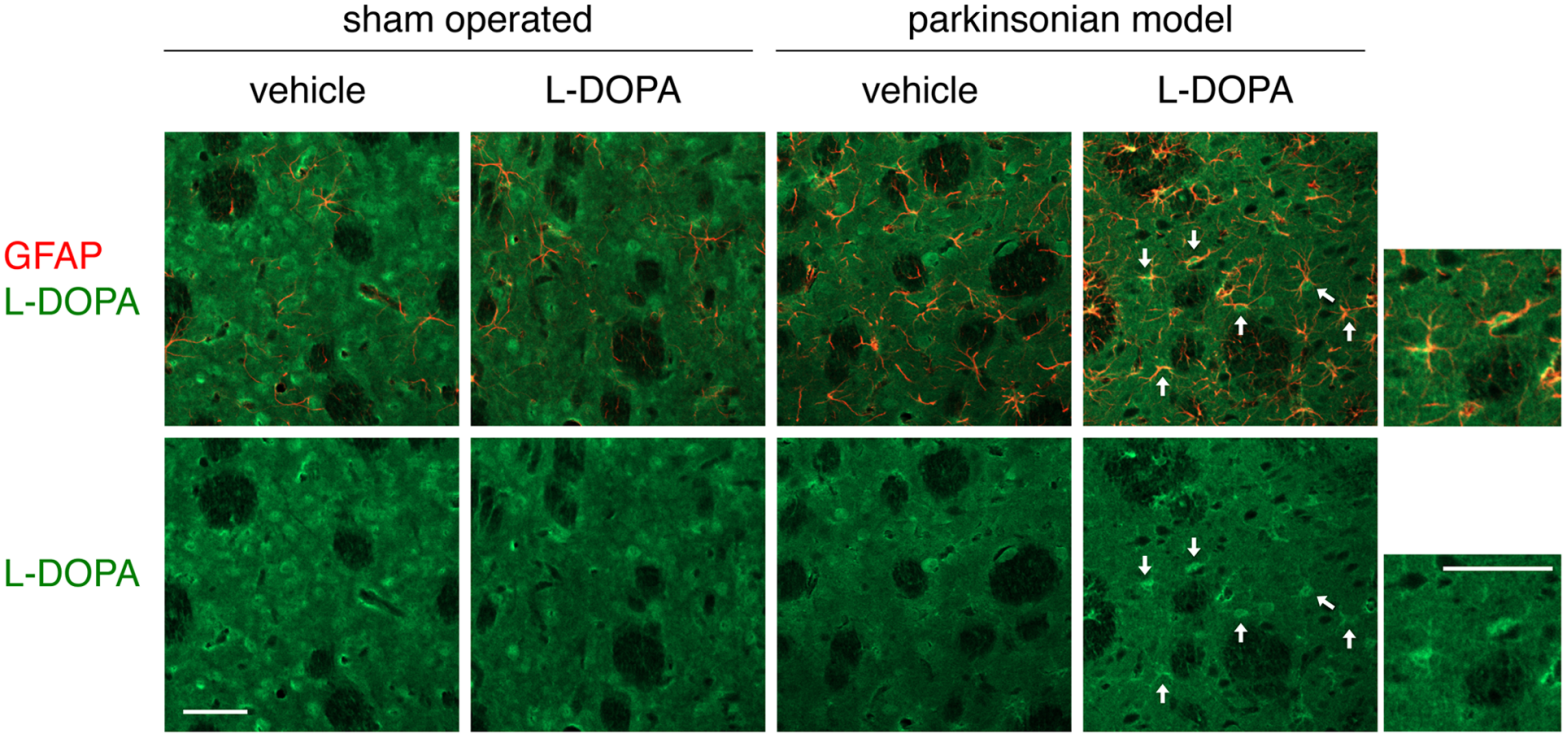
Expression of L-DOPA in reactive astrocytes in the lesioned side of the striatum of hemi-parkinsonian rats after L-DOPA administration. Confocal laser-scanning microscopic images of double immunofluorescein staining of L-DOPA (Alexa Fluor 488; green) and GFAP (Alexa Fluor 594; red) in the striatum of 6-OHDA-lesioned hemi-parkinsonian rats, and effects of repeated L-DOPA/carbidopa treatment (50/5 mg/kg/day, i.p.) for 7 days. Lower panels show immunoreactivity to L-DOPA. L-DOPA-immunopositive signals (white arrows) were observed in GFAP-labeled striatal astrocytes, especially on the lesioned side of PD models. Right small panels show higher magnification images of immunopositive signals in the L-DOPA-treated PD models. Scale bar = 50 µm.

**Figure 3:**
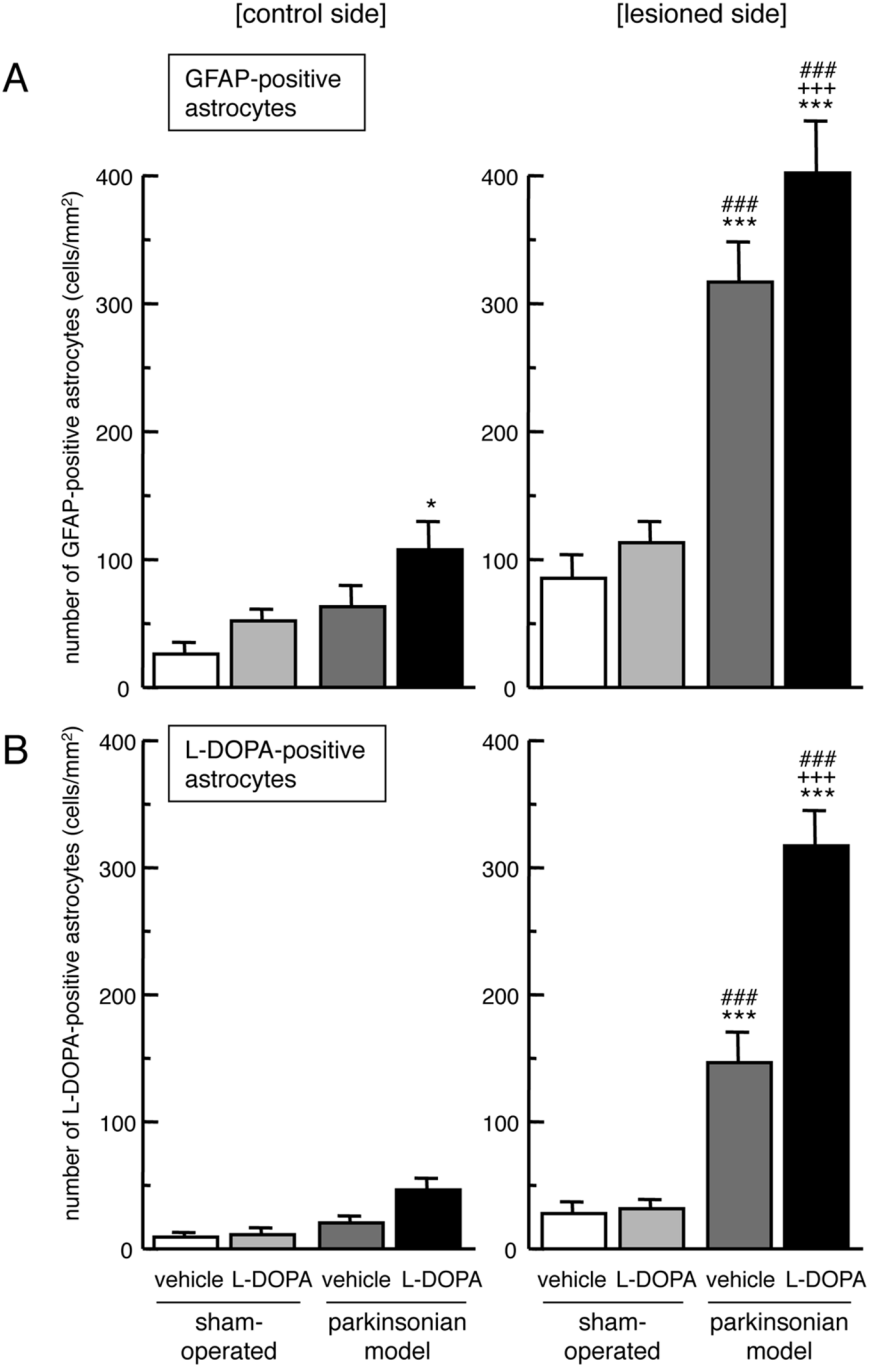
Changes in L-DOPA-immunoreactivity on reactive astrocytes in the striatum of hemi-parkinsonian rats after L-DOPA administration. The 6-OHDA-lesioned hemi-parkinsonian rats were treated with L-DOPA/carbidopa treatment (50/5 mg/kg/day, i.p.) for 7 days. The densities of GFAP-positive reactive astrocytes (A), and L-DOPA- and GFAP-double positive astrocytes (B) on both the control and lesioned sides of the striatum were evaluated by double immunostaining of L-DOPA and GFAP at 3 h after the final administration. Values are mean ± SEM of 8–10 mice. *p<0.05, ***p<0.001 vs. side-matched vehicle-treated sham-operated, +++p<0.001 vs. side-matched vehicle-treated parkinsonian group, ###p<0.001 vs. intact control side of each treated group.

We performed double immunostaining of GFAP and L-DOPA in the striatal sections of hemi-PD models that were repeatedly injected with L-DOPA/carbidopa (50/5 mg/kg/day, i.p.) for 7 days, and co-localization of both signals were analyzed by confocal laser-scanning microscopy. The 6-OHDA-lesioning markedly increased GFAP-positive reactive astrocytes in the striatum (Figs. 2 and 3). Some GFAP-positive astrocytes showed L-DOPA-immunoreactivity in the lesioned side of vehicle-treated PD models. Furthermore, the L-DOPA treatment apparently increased L-DOPA-immunoreactivity in GFAP-positive striatal astrocytes on the lesioned side in hemi-PD rats (Figs. 2 and 3).

### Expression of DA and DAT in the striatal astrocytes in hemi-parkinsonian models

**Figure 4:**
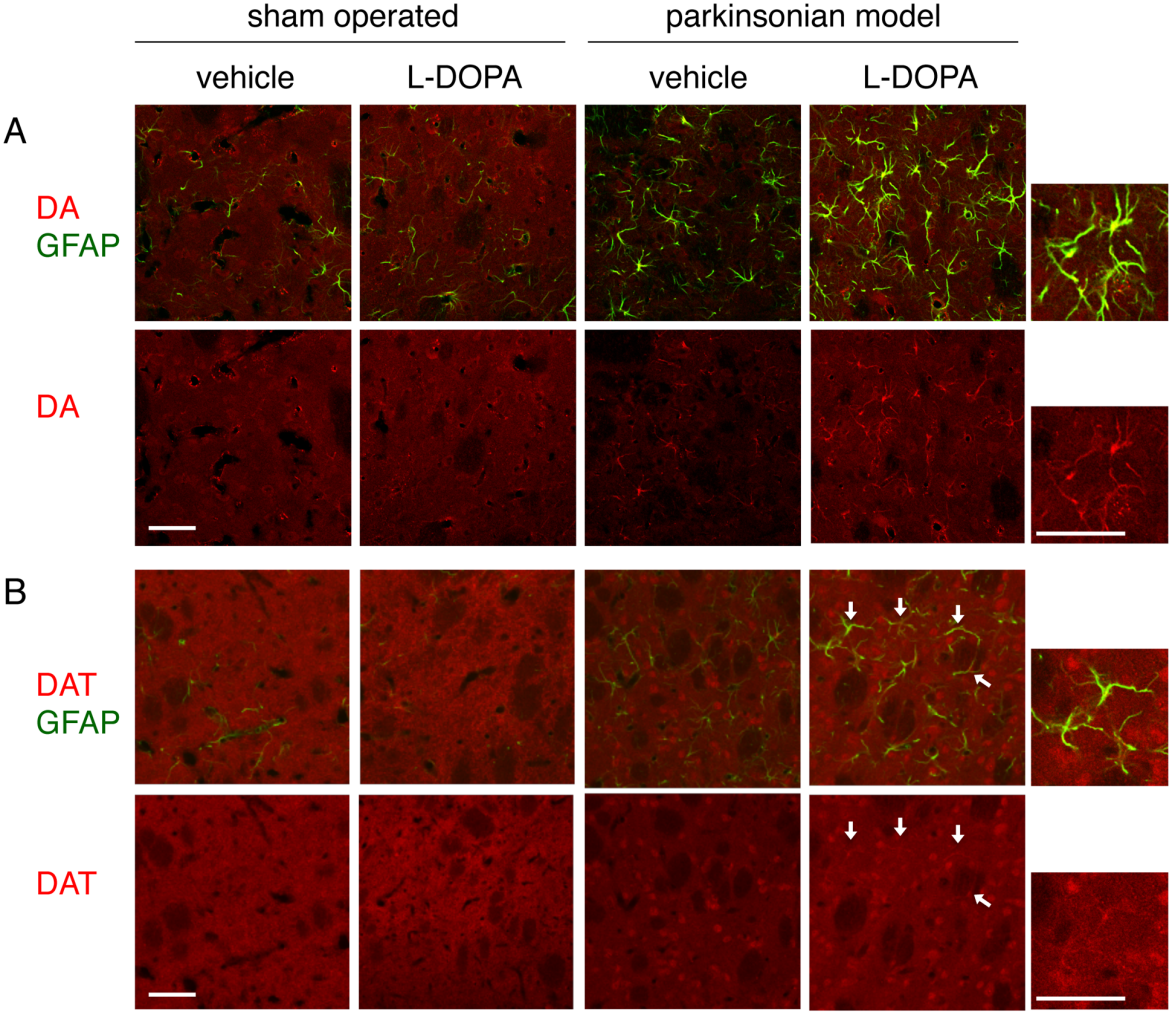
Expression of DA and DAT in reactive astrocytes in the lesioned side of the striatum of hemi-parkinsonian rats after L-DOPA administration. Confocal laser-scanning microscopic images of double immunofluorescein staining of DA (A; rhodamine; red) or DAT (B; rhodamine; red) and GFAP (FITC; green) in the striatum of 6-OHDA-lesioned hemi-parkinsonian rats, and effects of repeated L-DOPA/carbidopa treatment (50/5 mg/kg/day, i.p.) for 7 days. Lower panels show immunoreactivity to DA (A) and DAT (B). DA- and DAT-immunopositive signals (white arrows) were observed in GFAP-labeled striatal astrocytes, especially on the lesioned side of PD models. Right small panels show higher magnification images of immunopositive signals in the L-DOPA-treated PD models. Scale bar = 50 µm.

**Figure 5:**
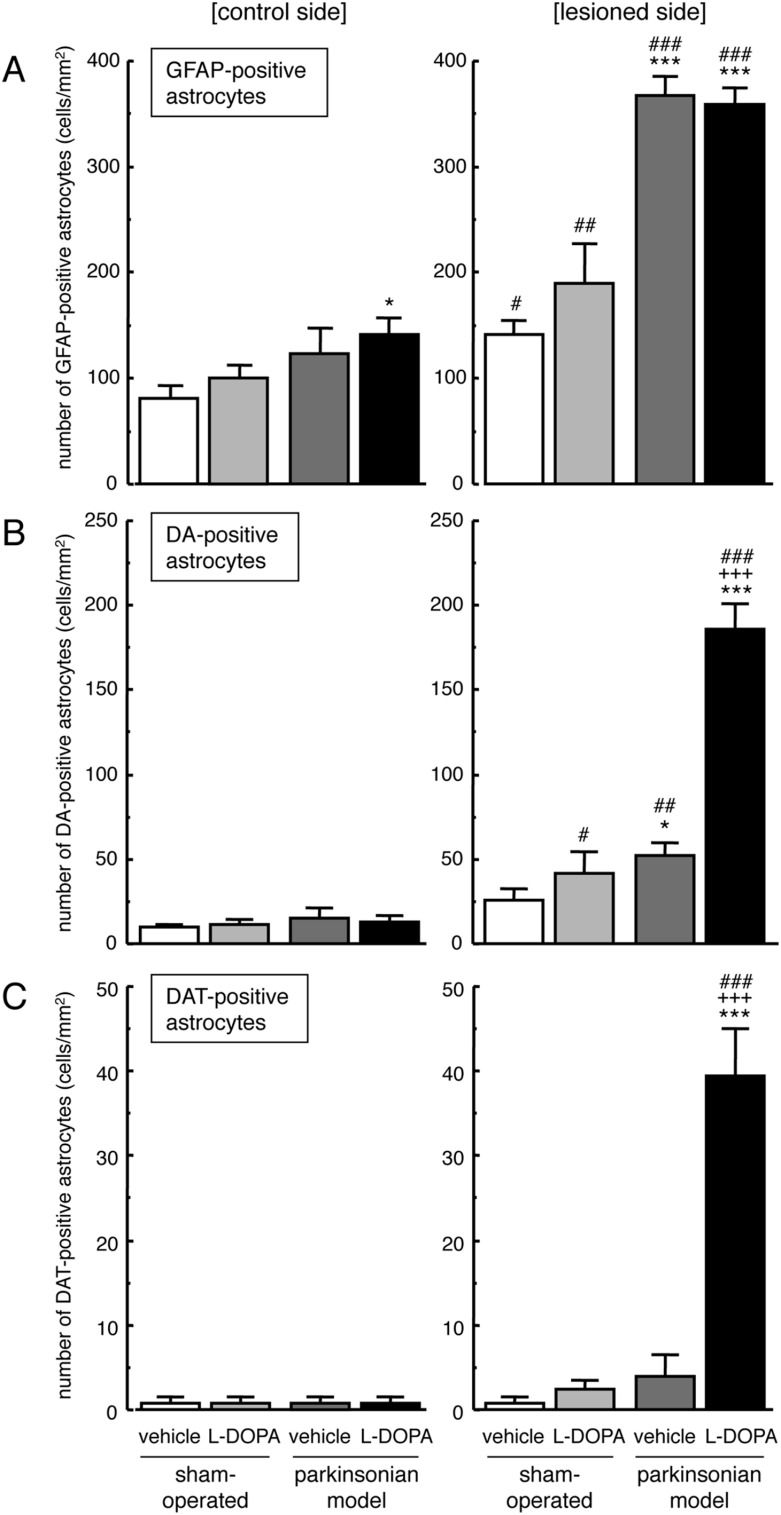
Changes in DA- and DAT-immunoreactivity on reactive astrocytes in the striatum of hemi-parkinsonian rats after L-DOPA administration. The 6-OHDA-lesioned hemi-parkinsonian rats were treated with L-DOPA/carbidopa treatment (50/5 mg/kg/day, i.p.) for 7 days. The densities of GFAP-positive reactive astrocytes (A), DA- and GFAP-double positive astrocytes (B) and DAT- and GFAP-double positive astrocytes (C) on both the control and lesioned sides of the striatum were evaluated by double immunostaining of DA/DAT and GFAP at 3 h after the final administration. Values are mean ± SEM of 6–8 mice. *p<0.05, ***p<0.001 vs. side-matched vehicle-treated sham-operated, +++p<0.001 vs. side-matched vehicle-treated parkinsonian group, #p<0.05, ##p<0.01, ###p<0.001 vs. intact control side of each treated group.

Examination of the lesioned side of the striatum in 6-OHDA-lesioned parkinsonian model rats showed apparent reduction of diffuse DA- and DAT-immunopositive signals, together with increased GFAP-immunopositive reactive astrocytes (Fig. 4). We examined the expression of DA and DAT in striatal astrocytes in hemi-parkinsonian rats that were repeatedly injected with L-DOPA/carbidopa (50/5 mg/kg/day, i.p.) for 7 days by confocal laser-scanning microscopy. Repeated L-DOPA injections induced marked expression of DAT and apparent DA-immunoreactivity in GFAP-positive reactive astrocytes on the lesioned side of the striatum in hemi-PD rats (Figs. 4 and 5). To a lesser extent, some GFAP-positive striatal astrocytes showed DA- and DAT-immunoreactivities in the lesioned side of vehicle-treated PD models.

### Expression of DA, DAT, LAT and AADC in cultured astrocytes

**Figure 6:**
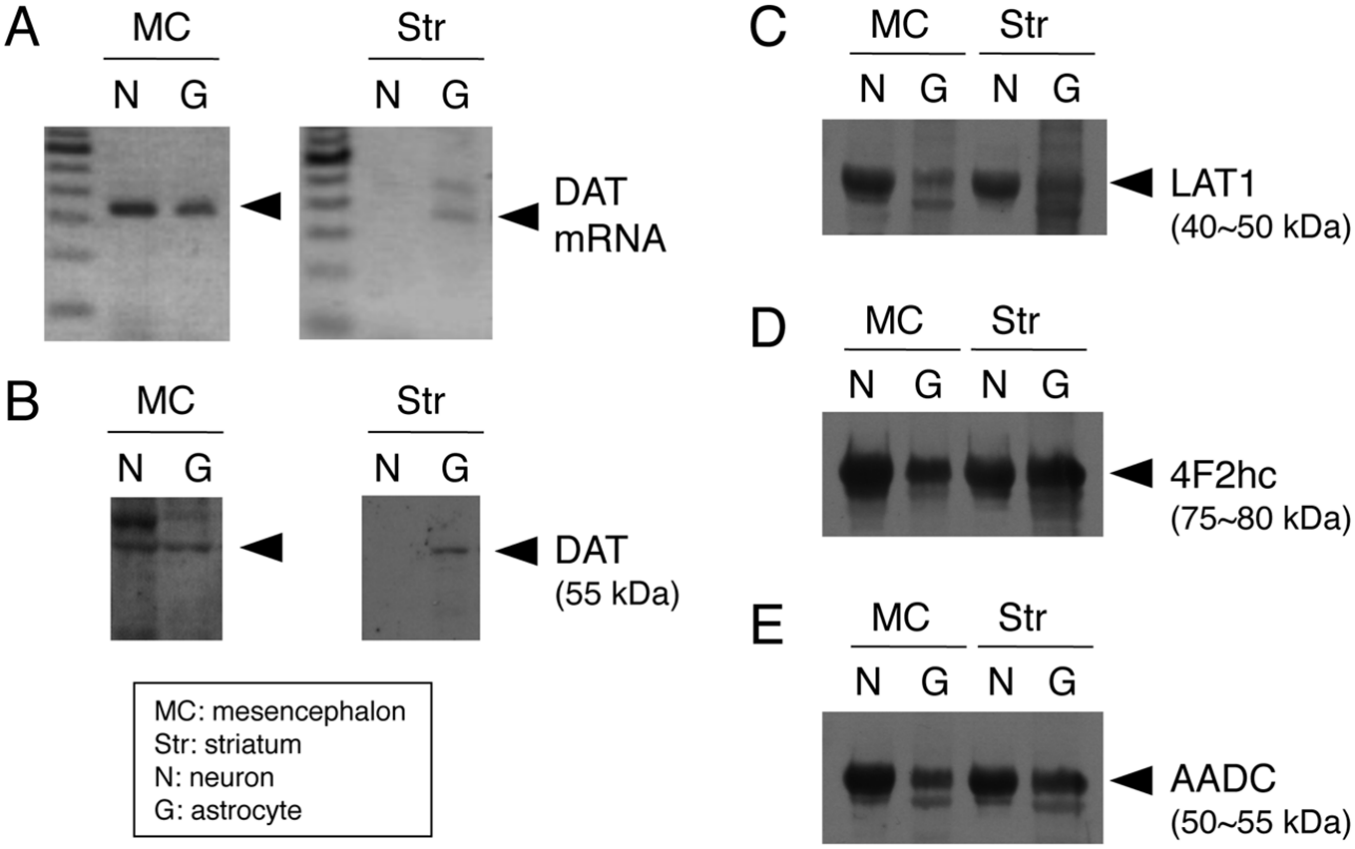
Expression of DAT, LAT1, its subunit and AADC in primary cultured neurons and astrocytes from rat mesencephalon and striatum. Detection of mRNA expression of DAT (A), protein expression of DAT (B), LAT1 (C), 4F2hc (D), and AADC (E) in primary cultured neurons (N) and astrocytes (G) from mesencephalon (MC) and striatum (Str) of rat embryos using RT-PCR assay (A) and western blot analysis (B–E).

To confirm the expression of molecules related to DA or L-DOPA uptake and metabolizing enzymes in astrocytes of basal ganglia, we performed RT-PCR to detect DAT mRNA and also western blot analysis for DAT, neutral amino acid transporter LAT of L-DOPA transporter and AADC, which catalyzes L-DOPA to DA, using primary cultured neuron-enriched cells or astrocytes from the mesencephalon or striatum. Although striatal neurons expressed neither DAT mRNA nor DAT protein, astrocytes from the mesencephalon and striatum expressed both DAT mRNA and protein, similar to mesencephalic neuron-enriched cells (Fig. 6A, 6B). LAT1 and 4F2hc (light and heavy chain of CD98 amino acid transporter, respectively) protein expression was detected not only in neurons of the basal ganglia but also in astrocytes of the mesencephalon and striatum (Fig. 6C, 6D). AADC protein expression was also detected in astrocytes of mesencephalic and striatal astrocytes, although the expression level in astrocytes was lower than in neurons (Fig. 6E).

### Uptake and metabolism of L-DOPA and DA in striatal astrocytes

**Figure 7:**
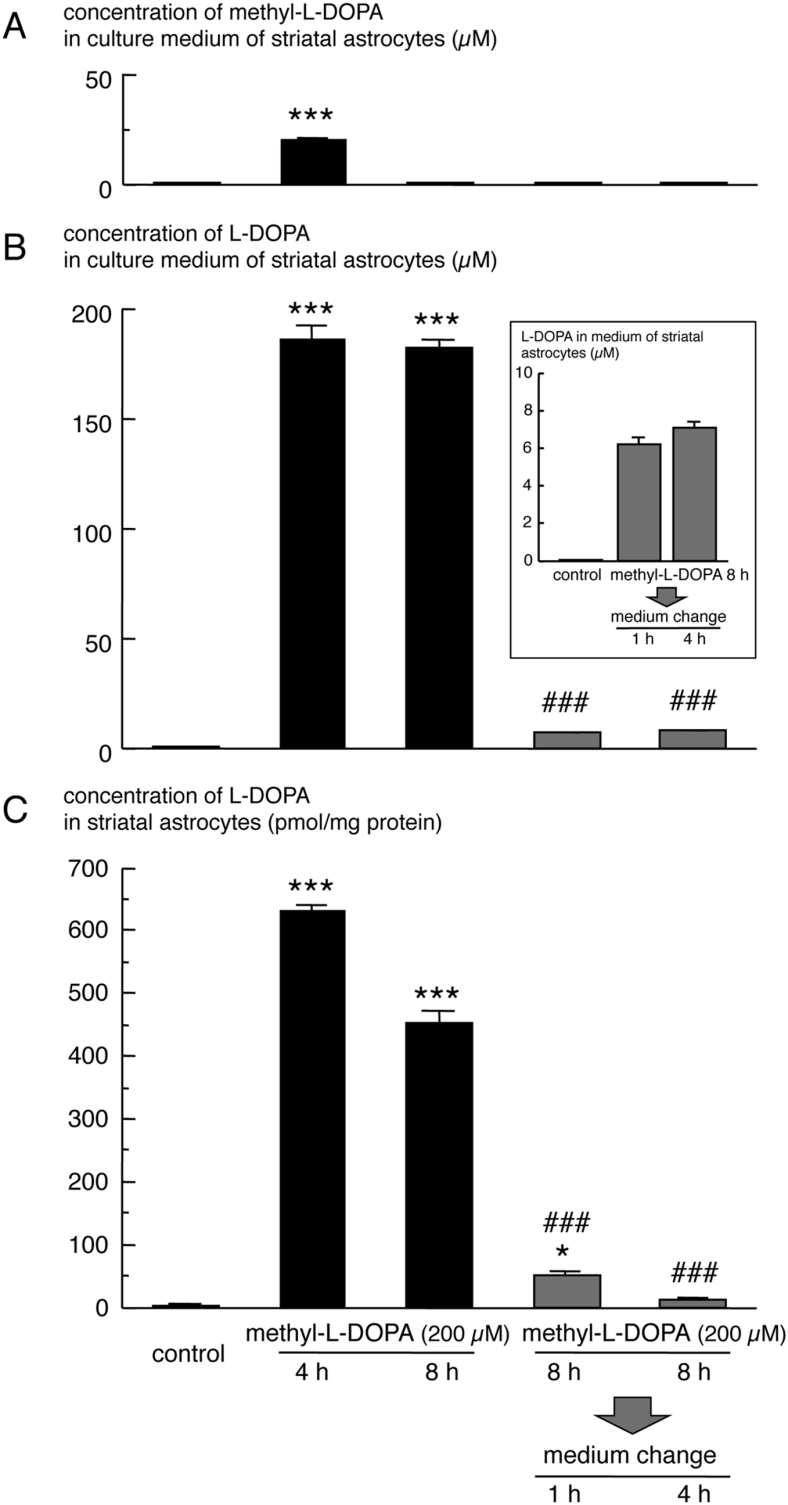
Changes in extracellular and intracellular L-DOPA concentration in methyl-L-DOPA-pretreated primary cultured striatal astrocytes after withdrawal of L-DOPA. Striatal astrocytes were treated with methyl-L-DOPA (200 µM) for 4 h or 8 h (filled bar). Furthermore, astrocytes pre-treated with methyl-L-DOPA for 8 h were incubated in fresh medium for 1 h or 4 h (gray bar). The concentration of methyl-L-DOPA (A) and L-DOPA (B) in culture media, and intracellular concentration of L-DOPA (C) in striatal astrocytes. Inset highlights the changes in L-DOPA concentration in fresh medium after removal of extracellular L-DOPA. Values are mean ± SEM (n = 4). *p<0.05, ***p<0.001 vs. control vehicle-treated, ###p<0.001 vs. 8-h methyl-L-DOPA-treated group.

We also measured the concentrations of L-DOPA, DA and their metabolite in primary cultured striatal astrocytes after exposure to methyl-L-DOPA or DA by HPLC, in order to clarify the uptake and metabolism of L-DOPA and DA in striatal astrocytes (Fig. 1, left, and Table 1). L-DOPA and DA were not detected in vehicle-pretreated astrocytes. However, marked elevation of intracellular DA and its metabolite DOPAC was noted in striatal astrocytes treated with DA for 4 h and 8 h (Table 1). Treatment with methyl-L-DOPA for 4 h induced marked increase in intracellular L-DOPA level in striatal astrocytes (Table 1 and Fig. 7C). This uptake of L-DOPA in striatal astrocytes was also noted at 8 h after methyl-L-DOPA treatment (Table 1 and Fig. 7C). On the other hand, methyl-L-DOPA in striatal astrocytes was undetectable either 4-h or 8-h methyl-L-DOPA-treated group. The concentration of methyl-L-DOPA in astrocyte culture media was rapidly decreased in a time-dependent manner after applying of methyl-L-DOPA (200 µM) and it declined to undetectable level at 8-h methyl-L-DOPA exposure (Fig. 7A), while the concentration of L-DOPA in culture media reached to maximum level (∼185 µM) at 4 h after the treatment (Fig. 7B). These indicate that applied methyl-L-DOPA was rapidly converted to L-DOPA in culture media, which was uptaken into astrocytes. Interestingly, however, DA and DOPAC were not detected in striatal astrocytes of either 4-h or 8-h methyl-L-DOPA-treated group (Table 1), despite the expression of AADC in striatal astrocytes (Fig. 6). When striatal astrocytes were cocultured with mesencephalic neurons, intracellular DA and DOPAC levels were increased in the astrocytes 4 h after methyl-L-DOPA treatment (Table 2).

**Table 1:** Concentrations of L-DOPA, dopamine and DOPAC in striatal astrocytes at 4 h or 8 h after methyl-L-DOPA or DA treatment.

	4 h		
	control	methyl-L-DOPA	DA
L-DOPA	ND	1349.7±48.1	ND
DA	ND	ND	302.0±41.9
DOPAC	ND	ND	3023.0±579.7
	8 h		
	control	methyl-L-DOPA	DA
L-DOPA	ND	1213.3±132.0#	ND
DA	ND	ND	372.9±23.5#
DOPAC	ND	ND	1204.9±127.5#

Striatal astrocytes were treated with methyl-L-DOPA, DA (200 µM) or control vehicle for 4 h or 8 h. The concentrations of L-DOPA, DA and DOPAC were measured by HPLC assay. ND: not detected. Data (pmol/mg protein) are presented as mean ± SEM (n = 4).

# p<0.001 vs. the corresponding 4-h treated group.

**Table 2:** Concentrations of L-DOPA, dopamine and DOPAC in striatal astrocytes cocultured with mesencephalic neurons at 4 h after methyl-L-DOPA treatment.

	4 h	
	control	methyl-L-DOPA
L-DOPA	ND	710.1±10.0
DA	1.1±0.03	17.9±0.2*
DOPAC	ND	0.5±0.05

Striatal astrocytes cocultured with mesencephalic neurons using cell insert were treated with methyl-L-DOPA (200 µM) or control vehicle for 4 h. The concentrations of L-DOPA, DA and DOPAC in striatal astrocytes were measured by HPLC assay. ND: not detected. Data (pmol/mg protein) are presented as mean ± SEM (n = 4).

*p<0.001 vs. control group.

Finally, to examine the release of uptaken L-DOPA in astrocytes, we measured changes in intracellular L-DOPA level in methyl-L-DOPA-pretreated striatal astrocytes after withdrawal of L-DOPA from the culture media (Fig. 1, middle, and Fig. 7). The elevated intracellular level of L-DOPA in cultured striatal astrocytes by pretreatment with methyl-L-DOPA for 8 h decreased rapidly after removal of extracellular L-DOPA (Fig. 7C). The level of L-DOPA in astrocytes reached the control level following their incubation in fresh normal medium for 4 h. On the other hand, the extracellular level of L-DOPA was increased at 1 h and 4 h after changing media (Fig. 7B).

## Discussion

In the present study, we examined possible uptake of L-DOPA- or DA- and the metabolic properties of striatal astrocytes using L-DOPA-administered hemi-parkinsonian rats and L-DOPA/DA-treated primary cultured striatal astrocytes. The main findings of this study are: (1) striatal astrocytes can uptake L-DOPA and DA, (2) the uptaken DA into striatal astrocytes is rapidly metabolized, (3) the uptaken L-DOPA is not converted to DA despite AADC expression in cultured striatal astrocytes, and (4) the intracellular level of L-DOPA in striatal astrocytes decreased rapidly after removal of extracellular L-DOPA.

It has been reported that certain receptors, channels and transporters for neurotransmitters are also expressed in astrocytes and are involved in neuron-astrocyte interaction [7], [8], [9], [10]. We reported previously that mRNAs and proteins of dopamine D1, D4 and D5 receptors are expressed in primary cultured astrocytes from the basal ganglia [22], suggesting that astrocytes as well as post-synaptic neurons can respond to DA. Furthermore, the reported expression of LAT and DAT in cortical and hippocampal astrocytes [11], [12], [13], [14], [15] suggests that striatal astrocytes can uptake L-DOPA and DA. In the present study, treatment with L-DOPA/carbidopa for 1 week induced apparent L-DOPA- and DA-immunoreactivities and marked DAT expression in reactive astrocytes on the lesioned side of the striatum in hemi-parkinsonian rats. In mixed cultures of neurons and astrocytes, DA treatment induced localized DA-immunoreactivity and increased DAT expression in reactive striatal astrocytes. Furthermore, primary cultured striatal astrocytes expressed DAT and LAT1 that can uptake DA and L-DOPA, respectively, and also expressed AADC that converts L-DOPA to DA. To our knowledge, this is the first report to show the expression of DAT and LAT in striatal astrocytes. The results suggest that striatal astrocytes can uptake L-DOPA and DA via LAT or DAT upon damage of dopaminergic neurons, and that such L-DOPA−/DA-uptake property of astrocytes may play an important role in L-DOPA metabolism in the damaged dopaminergic neural system.

To further understand the process of L-DOPA and DA uptake and metabolism in striatal astrocytes, the contents of L-DOPA, DA, and their metabolites in primary cultured striatal astrocytes were measured after L-DOPA/DA treatment. DA treatment increased the levels of DA and its metabolite DOPAC in striatal astrocytes, suggesting that released DA from the remaining DA neurons in patients with PD can be uptaken into astrocytes and rapidly metabolized. The level of L-DOPA in striatal astrocytes was markedly increased, but DA was not detected after L-DOPA exposure despite AADC expression in these cells. This finding suggests that L-DOPA can be uptaken into astrocytes while the uptaken L-DOPA is not converted to DA in astrocytes probably due to inactive AADC in striatal astrocytes. The rapid metabolism of the uptaken DA and minimal conversion of uptaken L-DOPA to DA in striatal astrocytes may reduce the availability of L-DOPA administered in damaged dopaminergic neurons. Therefore, the strong DA signal in reactive astrocytes on the lesioned side of the striatum in hemi-parkinsonian rats after long-term L-DOPA administration may be due to DA synthesized from the uptaken L-DOPA in surviving dopaminergic or serotonergic neurons. This idea is supported by the present result that L-DOPA treatment increased intracellular DA level in striatal astrocytes cocultured with mesencephalic neurons but not in the case of astrocyte alone.

The low conversion of uptaken L-DOPA to DA in striatal astrocytes raises the question of whether the uptaken L-DOPA in astrocytes is retained or released. To answer this question, we examined changes in intracellular L-DOPA level in L-DOPA-pretreated striatal astrocytes after withdrawal of L-DOPA from the culture media. Increased level of intracellular L-DOPA in cultured striatal astrocytes decreased rapidly to the control level after the removal of extracellular L-DOPA by changing media, and L-DOPA was detected in the fresh media. Taken together, the present results suggest that striatal astrocytes act as a reservoir for L-DOPA to uptake or release L-DOPA depending on extracellular L-DOPA concentration, but they cannot convert L-DOPA to DA.

## References

[1] Carta M, Carlsson T, Kirik D, Bjorklund A (2007) Dopamine released from 5-HT terminals is the cause of L-DOPA-induced dyskinesia in parkinsonian rats. Brain 130: 1819–1833. 17452372 10.1093/brain/awm082

[2] Lee J, Zhu WM, Stanic D, Finkelstein DI, Horne MH, et al (2008) Sprouting of dopamine terminals and altered dopamine release and uptake in Parkinsonian dyskinaesia. Brain 131: 1574–1587. 18487277 10.1093/brain/awn085

[3] Rylander D, Parent M, O’Sullivan SS, Dovero S, Lees AJ, et al (2010) Maladaptive plasticity of serotonin axon terminals in levodopa-induced dyskinesia. Ann Neurol 68: 619–628. 20882603 10.1002/ana.22097

[4] Tanaka H, Kannari K, Maeda T, Tomiyama M, Suda T, et al (1999) Role of serotonergic neurons in L-DOPA-derived extracellular dopamine in the striatum of 6-OHDA-lesioned rats. Neuroreport 10: 631–634. 10208602 10.1097/00001756-199902250-00034

[5] Megyeri K, Marko B, Sziray N, Gacsalyi I, Juranyi Z, et al (2007) Effects of 2,3-benzodiazepine AMPA receptor antagonists on dopamine turnover in the striatum of rats with experimental parkinsonism. Brain Res Bull 71: 501–507. 17259019 10.1016/j.brainresbull.2006.11.003

[6] Ogawa N, Tanaka K, Asanuma M (2000) Bromocriptine markedly suppress levodopa-induced abnormal increase of dopamine turnover in the parkinsonian striatum. Neurochem Res 25: 755–758. 10943992 10.1023/a:1007530720544

[7] Perea G, Navarrete M, Araque A (2009) Tripartite synapses: astrocytes process and control synaptic information. Trends Neurosci 32: 421–431. 19615761 10.1016/j.tins.2009.05.001

[8] Allaman I, Belanger M, Magistretti PJ (2010) Astrocyte-neuron metabolic relationships: for better and for worse. Trends Neurosci 34: 76–87. 10.1016/j.tins.2010.12.001 21236501

[9] Hamby ME, Sofroniew MV (2010) Reactive astrocytes as therapeutic targets for CNS disorders. Neurotherapeutics 7: 494–506. 20880511 10.1016/j.nurt.2010.07.003 PMC2952540

[10] Kimelberg HK, Nedergaard M (2010) Functions of astrocytes and their potential as therapeutic targets. Neurotherapeutics 7: 338–353. 20880499 10.1016/j.nurt.2010.07.006 PMC2982258

[11] Inyushin MY, Huertas A, Kucheryavykh YV, Kucheryavykh LY, Tsydzik V, et al (2012) L-DOPA uptake in astrocytic endfeet enwrapping blood vessels in rat brain. Parkinsons Dis 2012: 321406. 22888467 10.1155/2012/321406 PMC3409556

[12] Kim DK, Kim IJ, Hwang S, Kook JH, Lee MC, et al (2004) System L-amino acid transporters are differently expressed in rat astrocyte and C6 glioma cells. Neurosci Res 50: 437–446. 15567481 10.1016/j.neures.2004.08.003

[13] Tsai MJ, Lee EH (1996) Characterization of L-DOPA transport in cultured rat and mouse astrocytes. J Neurosci Res 43: 490–495. 8699535 10.1002/(SICI)1097-4547(19960215)43:4<490::AID-JNR10>3.0.CO;2-6

[14] Inazu M, Kubota N, Takeda H, Zhang J, Kiuchi Y, et al (1999) Pharmacological characterization of dopamine transport in cultured rat astrocytes. Life Sci 64: 2239–2245. 10374914 10.1016/s0024-3205(99)00175-7

[15] Inazu M, Takeda H, Ikoshi H, Uchida Y, Kubota N, et al (1999) Regulation of dopamine uptake by basic fibroblast growth factor and epidermal growth factor in cultured rat astrocytes. Neurosci Res 34: 235–244. 10576546 10.1016/s0168-0102(99)00053-x

[16] Pellegrino LJ, Pellegrino AS, Cushman AJ (1979) A stereotaxic atlas of the rat brain. New York and London: Plenum Press.

[17] Iwata-Ichikawa E, Kondo Y, Miyazaki I, Asanuma M, Ogawa N (1999) Glial cells protect neurons against oxidative stress via transcriptional up-regulation of the glutathione synthesis. J Neurochem 72: 2334–2344. 10349842 10.1046/j.1471-4159.1999.0722334.x

[18] Miyazaki I, Asanuma M, Kikkawa Y, Takeshima M, Murakami S, et al (2011) Astrocyte-derived metallothionein protects dopaminergic neurons from dopamine quinone toxicity. Glia 59: 435–451. 21264950 10.1002/glia.21112

[19] Xie GX, Jones K, Peroutka SJ, Palmer PP (1998) Detection of mRNAs and alternatively spliced transcripts of dopamine receptors in rat peripheral sensory and sympathetic ganglia. Brain Res 785: 129–135. 9526064 10.1016/s0006-8993(97)01394-2

[20] Asanuma M, Miyazaki I, Diaz-Corrales FJ, Kimoto N, Kikkawa Y, et al (2010) Neuroprotective effects of zonisamide target astrocyte. Ann Neurol 67: 239–249. 20225289 10.1002/ana.21885

[21] Diaz-Corrales FJ, Asanuma M, Miyazaki I, Miyoshi K, Ogawa N (2005) Rotenone induces aggregation of gamma-tubulin protein and subsequent disorganization of the centrosome: relevance to formation of inclusion bodies and neurodegeneration. Neuroscience 133: 117–135. 15893636 10.1016/j.neuroscience.2005.01.044

[22] Miyazaki I, Asanuma M, Diaz-Corrales FJ, Miyoshi K, Ogawa N (2004) Direct evidence for expression of dopamine receptors in astrocytes from basal ganglia. Brain Res 1029: 120–123. 15533323 10.1016/j.brainres.2004.09.014

